# A method for biomarker measurements in peripheral blood mononuclear cells isolated from anxious and depressed mice: β-arrestin 1 protein levels in depression and treatment

**DOI:** 10.3389/fphar.2013.00124

**Published:** 2013-09-26

**Authors:** Indira Mendez-David, Zeina El-Ali, René Hen, Bruno Falissard, Emmanuelle Corruble, Alain M. Gardier, Saadia Kerdine-Römer, Denis J. David

**Affiliations:** ^1^EA3544, Faculté de Pharmacie, Université Paris-SudChâtenay-Malabry, France; ^2^INSERM UMR 996, Faculté de Pharmacie, Université Paris-SudChâtenay-Malabry, France; ^3^Department of Neuroscience, Columbia UniversityNew York, NY, USA; ^4^Department of Psychiatry, Columbia UniversityNew York, NY, USA; ^5^INSERM U669, Département de Psychiatrie, Hôpital Bicêtre, Assistance Publique Hôpitaux de Paris, Université Paris-SudLe Kremlin-Bicêtre, France

**Keywords:** peripheral blood mononuclear cells, \boldsymbolβ-arrestin 1, anxiety, depression, mouse models, fluoxetine, biomarkers

## Abstract

A limited number of biomarkers in the central and peripheral systems which are known may be useful for diagnosing major depressive disorders and predicting the effectiveness of antidepressant (AD) treatments. Since 60% of depressed patients do not respond adequately to medication or are resistant to ADs, it is imperative to delineate more accurate biomarkers. Recent clinical studies suggest that β-arrestin 1 levels in human mononuclear leukocytes may be an efficient biomarker. If potential biomarkers such as β-arrestin 1 could be assessed from a source such as peripheral blood cells, then they could be easily monitored and used to predict therapeutic responses. However, no previous studies have measured β-arrestin 1 levels in peripheral blood mononuclear cells (PBMCs) in anxious/depressive rodents. This study aimed to develop a method to detect β-arrestin protein levels through immunoblot analyses of mouse PBMCs isolated from whole blood. In order to validate the approach, β-arrestin levels were then compared in na\"{\i}ve, anxious/depressed mice, and anxious/depressed mice treated with a selective serotonin reuptake inhibitor (fluoxetine, 18~mg/kg/day in the drinking water). The results demonstrated that mouse whole blood collected by submandibular bleeding permitted isolation of enough PBMCs to assess circulating proteins such as β-arrestin 1. β-Arrestin 1 levels were successfully measured in healthy human subject and na\"{\i}ve mouse PBMCs. Interestingly, PBMCs from anxious/depressed mice showed significantly reduced β-arrestin 1 levels. These decreased β-arrestin 1 expression levels were restored to normal levels with chronic fluoxetine treatment. The results suggest that isolation of PBMCs from mice by submandibular bleeding is a useful technique to screen putative biomarkers of the pathophysiology of mood disorders and the response to ADs. In addition, these results confirm that β-arrestin 1 is a potential biomarker for depression.

## INTRODUCTION

Elucidation of the neurobiological bases of depression and anxiety are significant challenges for today’s society. Mood disorders impact 7% of the world’s population and rank among the top 10 causes of disability (Kessler et al., 2005). Selective serotonin reuptake inhibitors (SSRIs) and serotonin and noradrenaline reuptake inhibitors (SNRIs) are the most commonly prescribed antidepressant (AD) drugs for major depressive disorders (MDD; Samuels et al., 2011). However, key questions about the molecular and cellular mechanisms underlying the effects of ADs remain unanswered. Approximately 60% of depression patients do not respond adequately or are resistant to these drugs (Samuels et al., 2011). Therefore, there are clear benefits of having valid, reliable, selective, and feasible biomarkers for MDD. Several studies have reported genome-wide expression changes associated with AD responses in MDD (Iga et al., 2007a, b; Belzeaux et al., 2010; Lakhan et al., 2010; Mamdani et al., 2011). However, candidate biomarkers that can accurately predict AD responses must be identified. While there are currently no specific markers that are considered “gold standards,” a few candidates have emerged. Peripheral/serum brain-derived neurotrophic factor (BDNF), insulin-like growth factor 1 (IGF-1), and cytokines may serve as biomarkers of MDD and treatment response (for review, see Schmidt et al., 2011).

Recently, a substantial body of evidence indicates that β-arrestins (β-arrestin 1 and 2), proteins that regulate G protein receptor coupling, play major roles in the pathophysiology of mood disorders and in the mechanisms underlying AD actions (Avissar et al., 2004; Schreiber and Avissar, 2004; Matuzany-Ruban et al., 2005; Beaulieu et al., 2008; David et al., 2009; Schreiber et al., 2009; Golan et al., 2010). The β-arrestin-signaling cascade has recently gained attention as a potential pre-clinical/clinical bridging biomarker for depressive states and treatment effects. In naïve rats, SSRI, SNRI, and non-selective reuptake inhibitor ADs significantly elevate β-arrestin 1 levels in the cortex and the hippocampus (Avissar et al., 2004; Beaulieu and Caron, 2008; Beaulieu et al., 2008; David et al., 2009). Similarly, β-arrestin 1 expression is decreased in the hypothalamus and hippocampus in anxious/depressed mice exposed to glucocorticoid elevation, and is restored by chronic fluoxetine treatment (David et al., 2009). Moreover, β-arrestin 1 and 2 signaling is involved in mediating the response to fluoxetine and lithium (Beaulieu et al., 2008; David et al., 2009).

Clinical data from Avissar et al. (2004) suggest that β-arrestin 1 mRNA and protein levels are highest in peripheral blood leukocytes of MDD patients. Therefore, β-arrestin 1 may be a putative candidate biochemical marker in clinical practice for depressive pathophysiology and the response to ADs (for review, see Schreiber et al., 2009). β-Arrestin mRNA levels and β-arrestin 1 protein levels in mononuclear leukocytes of untreated patients with MDD are lower than the levels found in healthy subjects. Furthermore, reduced levels of β-arrestin 1 protein and mRNA are significantly correlated with the severity of depressive symptoms (Avissar et al., 2004; Schreiber et al., 2009). However, the low β-arrestin 1 protein and mRNA levels are alleviated by AD treatment. Therefore, these low levels can predict clinical improvement (Avissar et al., 2004; Golan et al., 2010).

These clinical data suggest that assessment of β-arrestin 1 levels may prove useful for diagnosing depression with high sensitivity and specificity (Golan et al., 2013). This hypothesis must first be validated in animal models of anxiety–depression. Most of the current understandings of mood disorders and AD activities are based on studies performed on animal models of anxiety–depression (Belzung and Lemoine, 2011). No animal studies have investigated whether β-arrestin 1 protein levels in peripheral blood mononuclear cells (PBMCs) area marker of the pathophysiology of depression and the AD response. However, if PBMCs can be successfully used to define biomarkers, they provide a system of circulating cells that can be easily collected from patients and monitored to predict therapeutic responses.

In this study, we developed a method to measure and assess circulating proteins (such as β-arrestin 1 in PBMCs) that are collected through submandibular bleeding from unanesthetized animals. Furthermore, we examined whether changes in β-arrestin 1 levels in mouse PBMCs were observed in a model of anxiety/depression (David et al., 2009; Guilloux et al., 2011; Rainer et al., 2012b), and whether these levels could be corrected by chronic treatment with the SSRI fluoxetine.

## EXPERIMENTAL PROCEDURES

### SUBJECTS

Adult male C57BL/6Ntac mice were purchased from Taconic Farms (Lille Skensved, Denmark). All mice were 7–8 weeks old, weighed 23–25 g at the beginning of the treatment and were maintained on a 12L:12D schedule (lights on at 0600 hours). The mice were group-housed with each cage containing five animals. Food and water were provided *ad libitum*. All testing were conducted in compliance with the laboratory animal care guidelines and with protocols approved by the Institutional Animal Care and Use Committee (Council directive # 87-848, October 19, 1987, Ministère de l’Agriculture et de la Forêt, Service Vétérinaire de la Santé et de la Protection Animale, permissions # 92-256B to Denis J. David).

### DRUGS

Corticosterone (4-pregnen-11b-DIOL-3 20-DIONE 21-hemi-succinate from Sigma (Sigma-Aldrich, Saint-Quentin-Fallavier, France) was dissolved in 0.45% hydroxypropyl-β-cyclodextrin (Sigma-Aldrich, Saint-Quentin-Fallavier, France). Fluoxetine hydrochloride (18 mg/kg/day in the drinking water) was purchased from Anawa Trading (Zurich, Switzerland).

### ISOLATION OF HUMAN AND MOUSE PERIPHERAL BLOOD MONONUCLEAR CELLS

#### Collection of human blood and isolation of peripheral blood mononuclear cells

**FIGURE 1:**
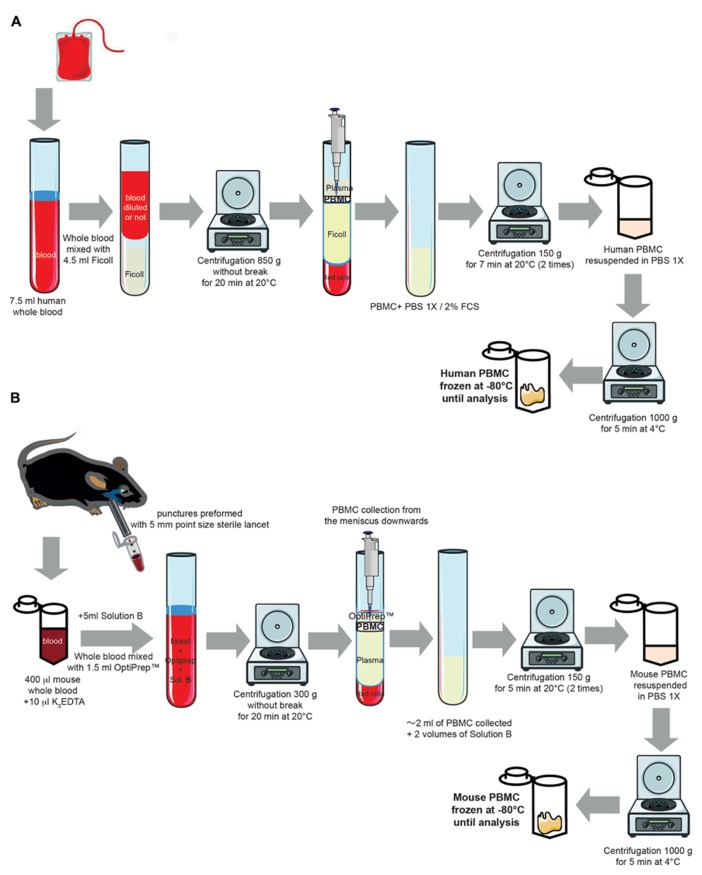
**Experimental protocol for isolating human and mouse peripheral blood mononuclear cells from whole blood. (A)** Cartoon representing the different steps for isolating human PBMC from whole circulating blood (for full details of the method, see *Blood collection and Peripheral blood mononuclear cells Isolation in human* from the Section “Experimental Procedures”). Some elements of this Figure were produced using Servier Medical Art image bank (). **(B)** Cartoon representing the different steps for isolating mouse PBMC from whole circulating blood (for full details of the method, see *Blood collection and Peripheral blood mononuclear cells Isolation in mouse* from the Section “Experimental Procedures”). Some elements of this figure were produced using Servier Medical Art image bank ().

Peripheral blood mononuclear cells were purified from 7.5 ml of human whole circulating blood obtained from Etablissement Français du Sang (Ivry-Sur-Seine, France) through density centrifugation (850 *g* at 20°C for 20 min) using a Ficoll gradient (PAA Laboratories GmbH, Pashing, Austria; **Figure 1A**). This centrifugation separated lymphocytes, monocytes, and plasma. The PBMC layers were carefully removed from the tube and transferred to a new 50 ml conical tube. The PBMCs were then washed twice (1 min each) with 1× phosphate-buffered saline (PBS)/fetal calf serum (FCS, 2%). After centrifugations (150 *g* at 20°C for 7 min), the cells were resuspended in the appropriate volume of 1× PBS. The human PBMCs were then recovered with a final centrifugation (1,000 *g* at 4°C for 5 min) and were stored at -80°C.

#### Collection of mouse blood and isolation of peripheral blood mononuclear cells

Blood was collected from unanesthetized mice as previously described (Golde et al., 2005; Joslin, 2009). In compliance with the laboratory animal care guidelines, approximately 0.4 ml of blood per mouse was collected in K_3_EDTA tubes with a submandibular bleeding procedure. Five millimeters point size sterile lancets (MediPoint, Mineola, NY, USA; **Figure 1B**) were used to puncture the location where the orbital vein and the submandibular vein join to form the jugular vein (Joslin, 2009). A light pressure with dry gauze was applied to the punctured area for hemostasis. Separation and extraction of PBMCs were performed using an iodixanol mixer technique (Ford and Rickwood, 1990). Mouse PBMCs were purified from whole blood by density centrifugation (300 *g* at 20°C for 30 min) using solution B (see **Table 1** for preparation) of the OptiPrep^TM^ gradient solution (Sigma-Aldrich, Saint-Quentin-Fallavier, France). Specifically, the OptiPrep^TM^ gradient solution was used to separate blood into PBMC and plasma layers with centrifugation. The PBMC layers were then carefully removed from the tube and transferred to a new 50 ml conical tube. The PBMCs were then washed twice with solution B (1 min each). After another centrifugation (150 *g* at 20°C for 7 min) and two washing steps (1 min each), mouse PBMCs were recovered with a final centrifugation (1,000 *g* at 4°C for 5 min) and were stored at -80°C.

**Table 1:** Solution used to prepare peripheral blood mononuclear cells from mouse whole blood.

	OptiPrep^TM^ density gradient medium	Tricine-buffered saline (TBS)	Solution B
Solutions	D1556-250ML (Sigma-Aldrich, France)	0.85% NaCl, 10 mM; Tricine-NaOH, pH 7.4 (*Tricine as 100 mM stock solution at 4°C*; 1.79 g/100 ml water)	Dissolve 0.85 g NaCl in 50 ml water; add 10 ml of Tricine stock; adjust to pH 7.4 with 1 M NaOH and make up to 100 ml

### β-ARRESTIN 1 LEVELS IN HUMAN AND MOUSE PERIPHERAL BLOOD MONONUCLEAR CELLS

#### Protein extraction from peripheral blood mononuclear cells and immunoblots

Peripheral blood mononuclear cells were thawed and homogenized with cell lysis buffer containing [20 mM Tris pH 7.4, 137 mM NaCl, 2 mM ethylenediaminetetraacetic acid (EDTA) pH 7.4, 1% Triton X-100, 25 mM β-glycerophosphate, 1 mM phenylmethylsulfonyl fluoride (PMSF), 10 μg/ml aprotinin, 10 μg/ml leupeptin, and 10 μg/ml pepstatin and 100 mM orthovanadate], were incubated on ice for 20 min, were then subjected to centrifugation at 21,130 *g* at 4°C for 20 min. Protein concentrations were quantified using a BCA Protein Assay Kit (Pierce Biotech-nology).

#### β-Arrestin 1 level measurements with immunoblot analyses

**FIGURE 2:**
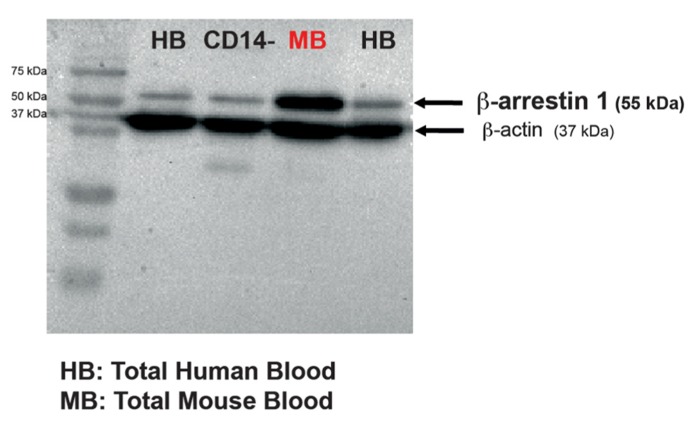
**β-Arrestin 1 is measurable in both human and mouse peripheral blood mononuclear cells obtained from a low collection volume of fresh blood.** Representative western blot of β-arrestin 1 levels in PBMCs isolated either from CD14^-^ human cells, human or naïve mouse whole blood. In each blot, 30 μg of total protein were run. β-Actin was used as a control.

**FIGURE 3:**
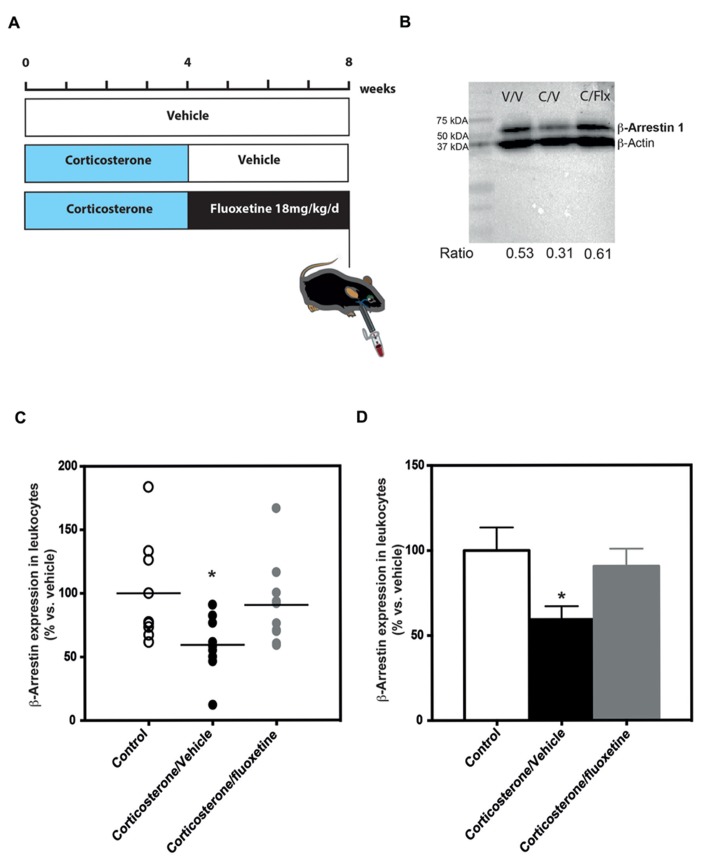
**Chronic fluoxetine treatment (18 mg/kg/day for 28 days) restored β-arrestin 1 levels in the peripheral blood mononuclear cells from anxio/depressive mice treated with chronic corticosterone treatment. (A)** Experimental timeline used to measure β-arrestin 1 levels in peripheral blood mononuclear cells from naïve or anxio-depressive mouse submandibular bleeding chronically treated or not with fluoxetine during 28 days (18 mg/kg/day in the drinking water). **(B)** Representative western blot of β-arrestin 1 levels in peripheral blood mononuclear cells isolated from naïve (vehicle/vehicle, V/V), corticosterone (35 μg/ml/day; corticosterone/vehicle, C/V) or corticosterone/fluoxetine (18 mg/kg/day; corticosterone/fluoxetine, C/F) mouse whole blood. In each blot, 30 μg of protein from mouse PBMC were run. The densitometry values for each band allowed the calculation of a ratio: optical density for β-arrestin 1/optical density β-actin value. **(C,D)** Scatterplot of the individual effects **(C)** or bar charts of mean ± SEM of the effects **(D)** of a chronic administration (28 days) treatment with fluoxetine on β-arrestin 1 levels in the peripheral blood mononuclear cells from mice treated with chronic corticosterone in comparison to untreated animals. Data are expressed in percentage normalized to vehicle/vehicle expression; *n* = 9–10). *p < 0.05, *versus* control vehicle/vehicle group.

Equal amounts of proteins were separated by 10% sodium dodecyl sulfate-polyacrylamide gel electrophoresis (SDS-PAGE) and transferred to polyvinylidene difluoride (PVDF) membranes (Amersham Biosciences, Les Ulis, France). The membranes were then incubated overnight with a primary mouse monoclonal anti-β-arrestin 1 antibody (#610551, BD Biosciences Pharmingen, France; 1:100). In order to ensure that equal amounts of total protein (30 μg) were loaded in each lane, β-actin protein levels were also assessed [β-actin (C4) horseradish peroxidase (HRP), Santa Cruz Biotechnology, Germany, 1:10,000]. Immune complexes were detected using appropriate peroxide-conjugated secondary antibodies and a chemiluminescent reagent kit (Pierce Biotechnology). Immunoblot quantifications were performed by densitometric scanning with Image Lab Software (Bio-Rad). Signals were in the linear range. The densitometry values were normalized against the β-actin values (**Figures 2** and **3**).

### CORTICOSTERONE MODEL AND TREATMENT

The dose and duration of corticosterone treatment (CORT model) were selected based on previous studies (David et al., 2009; Guilloux et al., 2011; Hache et al., 2012; Rainer et al., 2012a, b). Exposure to chronic corticosterone results in a phenotype that is similar to a chronic stress phenotype, including a deterioration of the coat state and anxiety/depression-related behaviors. At the end, a higher emotionality score is observed (Guilloux et al., 2011). Corticosterone (35 μg/ml/day, equivalent to about 5 mg/kg/day) or vehicle (0.45% β-cyclodextrin, β-CD) were available to mice *ad libitum* in the drinking water in opaque bottles. Corticosterone-treated water was changed every 3 days to prevent degradation. Group-housed mice were also treated with the SSRI fluoxetine (18 mg/kg/day) for the last 4 weeks of the experiment (see the experimental protocol on **Figure 3A**).

### STATISTICAL ANALYSIS

β-Arrestin 1 levels were quantified and then expressed with a scatterplot or as mean ± SEM normalized to vehicle levels. Data were analyzed using Prism 6.0 software (GraphPad, La Jolla, USA). One-way ANOVAs were used to evaluate data when appropriate. Significant main effects were further analyzed by Fisher’s *post hoc* test. Statistical significance was set at *p* < 0.05.

## RESULTS

### β-ARRESTIN 1 IS DETECTED IN HUMAN AND MOUSE PBMC

We first collected blood in order to assess whether β-arrestin 1 could be detected. Single-use lancets were used for submandibular bleeding and permitted drawing of ~0.4 ml of blood without the use of anesthesia (Golde et al., 2005). The mouse PBMCs were lysed and subjected to immunoblotting. A ~55 kDa band that corresponded to the molecular weight of β-arrestin 1 protein was detected with a monoclonal antibody against mouse anti-β-arrestin 1 that is known to detect human β-arrestin 1 (Avissar et al., 2004; Matuzany-Ruban et al., 2005; Golan et al., 2013; **Figure 2**). Therefore, this method of PBMC isolation from fresh mouse blood successfully permitted measurements of β-arrestin 1 levels. This method can potentially be used to investigate levels of other proteins as well. Lysates of human total PBMCs and CD14 negative PBMC fraction cells (CD14^-^) were used as positive controls. In addition, we were also able to detect β-arrestin 1 in human PBMCs isolated from low fresh circulating blood volume (7.5 ml) of healthy adult donors obtained from Etablissement Français du Sang (**Figure 2**). To our knowledge, this is the first study to detect β-arrestin 1 in this fashion.

Next, we decided to quantify β-arrestin 1 levels in PBMCs isolated from C57BL/6Ntac mice exposed to chronic corticosterone (David et al., 2009; Rainer et al., 2012b) that was given either alone or in combination with the SSRI fluoxetine (18 mg/kg/day; **Figure 3A**).

### CHRONIC FLUOXETINE TREATMENT NORMALIZES β-ARRESTIN 1 EXPRESSION IN PBMC ISOLATED FROM ANXIOUS/DEPRESSIVE-LIKE MICE

In mouse PBMCs isolated from blood of mice treated chronically with corticosterone (35 μg/ml/day), we found that β-arrestin 1 levels were significantly lower (-41%; 59% of expression compared to 100% in the control group) than the levels in naïve animals [one-way ANOVA, *F*(2,25) = 3.81; *p < 0.05; **Figures 3C, D**]. Interestingly, a 4-week treatment with the SSRI fluoxetine normalized these β-arrestin 1 expression levels so that they were not significantly different than the levels observed in naïve animals (**Figures 3C, D**).

## DISCUSSION

We developed a new method to assess circulating proteins such as β-arrestin 1 through immunoblot analyses of mouse PBMCs isolated from whole blood. We showed significantly reduced β-arrestin 1 levels in PBMCs from anxious/depressed mice. These decreased β-arrestin 1 expression levels were restored to normal levels with chronic fluoxetine treatment.

### PBMCs WERE ISOLATED FROM UNANESTHETIZED MICE

A recent review from Duman’s group highlighted the need to develop a biomarker panel for depression. This biomarker panel should profile diverse peripheral factors that together will provide a biological signature of MDD subtypes and predict treatment response (Schmidt et al., 2011). Assessing peripheral protein levels in PBMCs is an attractive method because PBMCs are circulating cells that can be easily collected and monitored. Previous studies demonstrated that PBMCs can be isolated from mouse blood to assess immunological responses (Fuss et al., 2009). However, to our knowledge this is the first study to collect PBMCs from circulating blood of unanesthetized animals. Single-use lancets were used for submandibular bleeding. This method permitted PBMCs to be collected from peripheral blood circulation in living and unanesthetized mice. Thus, submandibular bleeding is a useful method to screen putative biomarkers of the pathophysiology of mood disorders and the response to ADs. This technique can be easily performed multiple times in the same animals and can be used with other rodent species such as rats.

### β-ARRESTIN 1 PROTEIN LEVELS CAN BE MEASURED IN MOUSE AND HUMAN PBMCs

We measured β-arrestin 1 protein levels to determine whether mouse PBMCs are useful biological materials to screen biomarkers for MDD pathophysiology and the AD response. Over the last decade, several G protein receptor-related genes such as β-arrestins were found to be involved in the pathophysiology of mood disorders (Schreiber and Avissar, 2004; Beaulieu et al., 2008; David et al., 2009). Numerous data from clinical studies support the importance of measuring β-arrestin 1 levels as a peripheral biomarker of the pathophysiology of mood disorders and predicting the AD response (Avissar et al., 2004; Schreiber et al., 2009; Golan et al., 2013). However, no previous study demonstrated *ex vivo* measurements of β-arrestin 1 levels in leukocytes isolated from whole blood to compare levels between naïve and anxious/depressed rodents. In addition, this is the first study to assess β-arrestin 1 by immunoblot in human and in mouse leukocytes simultaneously by using the same monoclonal antibody.

In the human experiments, we were able to recover PBMCs from 7.5 ml of whole circulating blood from healthy volunteers. Previous studies showed that larger amounts of blood were needed for the detection of β-arrestin 1 in human leucocytes (Avissar et al., 2004; Matuzany-Ruban et al., 2005; Golan et al., 2013). Here, 7.5 ml was sufficient to acquire 30 μg of PBMC lysate for immunoblotting (**Figure 2**).

Avissar et al. (2004) demonstrated that β-arrestin 1 levels were elevated by chronic ADs in rat cortex and hippocampus. However, by contrast with their human study, they did not provide data showing that β-arrestin 1 levels in rat PBMCs are affected by chronic AD treatment (Avissar et al., 2004). Therefore, we also compared β-arrestin 1 levels in PBMCs of anxious/depressed mice before and after chronic AD treatment (**Figure 3**).

### β-ARRESTIN 1 IS A PREDICTIVE MARKER OF THE PATHOPHYSIOLOGY OF DEPRESSION AND THE ANTIDEPRESSANT RESPONSE

To induce an anxious/depression-related phenotype, we utilized a chronic corticosterone treatment that results in hallmark characteristics of anxiety and depression (for review, see David et al., 2009; Mendez-David et al., 2013). In order to delineate a panel of biomarkers of the pathophysiology and the treatment of depression, it is first essential to screen putative candidates in a model of anxiety/depression. β-Arrestin 1 protein levels in leukocytes were reduced when mice were exposed to chronic corticosterone. As found in previous human studies (Matuzany-Ruban et al., 2005; Golan et al., 2013), these reduced β-arrestin 1 levels were alleviated by AD treatment.

### LIMITATIONS OF THE STUDY

Measuring protein levels in mouse PBMCs at several time points is a powerful technique that can be used to reveal potential biomarkers for the pathophysiology of depression and the AD response. However, this study has some limitations that must be considered when interpreting the current findings. For example, it is important to distinguish diagnostic biomarkers from treatment biomarkers (Schmidt et al., 2011). This study does not address this difference. Further studies are required to assess whether β-arrestin 1 is a reasonable biomarker for diagnostic and/or drug treatments. A study that compares peripheral levels of β-arrestin 1 in stressed animals before and after AD treatment could definitively address this question. It also may be interesting to study whether there is a correlation between β-arrestin 1 levels and the severity of the anxio/depressive state (Guilloux et al., 2011). Moreover, disease conditions are most often signified by the dysregulation of complex biological pathways involving multiple key factors (Dudley and Butte, 2009). Thus, it is unlikely that β-arrestin 1 alone will be a sufficient diagnostic and treatment biomarker. However, mouse PBMCs might provide useful material to screen a panel of biomarkers and to provide biological signatures of MDD and AD treatments. Finally, in our study, β-arrestin 1 levels were measured using western blots, which is a semi-quantitative method of evaluating protein levels. The development of an enzyme-linked immunosorbent assay (ELISA) to assess β-arrestin 1 levels would provide a more quantitative method.

## CONCLUSION

In this study, we demonstrated that PBMCs isolated from a small volume of whole blood in unanesthetized mice using a submandibular bleeding method may provide a useful biological tool to assess circulating proteins. This method will permit future studies to screen potential biomarkers for the pathophysiology of depression and AD responses. We also confirmed that measurements of β-arrestin 1 levels in PBMCs may serve as a biochemical marker of depression in humans (Avissar et al., 2004). Overall, we developed a powerful tool for translational studies that can easily be used to assess proteins measurements and to provide a biological signature of treatment response. Identification of a biological signature could predict the effectiveness of ADs (Fuss et al., 2009).

## Conflict of Interest Statement

The authors declare that the research was conducted in the absence of any commercial or financial relationships that could be construed as a potential conflict of interest.

## AUTHOR CONTRIBUTIONS

Indira Mendez-David, Alain M. Gardier, René Hen, Saadia Kerdine-Römer, and Denis J. David designed research; Indira Mendez-David and Zeina El-Ali performed research and draw **Figure 2**; Indira Mendez-David analyzed data; Indira Mendez-David, Saadia Kerdine-Römer, and Denis J. David wrote the manuscript. Indira Mendez-David, Zeina El-Ali, René Hen, Emmanuelle Corruble, Bruno Falissard, Alain M. Gardier, Saadia Kerdine-Römer, and Denis J. David contributed to the preparation of the manuscript.
